# Amitriptyline functionally antagonizes cardiac H_2_ histamine receptors in transgenic mice and human atria

**DOI:** 10.1007/s00210-021-02065-7

**Published:** 2021-02-24

**Authors:** Joachim Neumann, Maximilian Benedikt Binter, Charlotte Fehse, Margaréta Marušáková, Maren Luise Büxel, Uwe Kirchhefer, Britt Hofmann, Ulrich Gergs

**Affiliations:** 1 Institut für Pharmakologie und Toxikologie, Medizinische Fakultät, Martin-Luther-Universität Halle-Wittenberg, D-06097 Halle, Germany; 2 Department of Pharmacology and Toxicology, Faculty of Pharmacy, Comenius University in Bratislava, Bratislava, Slovakia; 3 Institut für Pharmakologie und Toxikologie, Medizinische Fakultät, Westfälische Wilhelms-Universität, Domagkstr. 12, D-48149 Münster, Germany; 4 Cardiac Surgery, Medizinische Fakultät, Martin-Luther-Universität Halle-Wittenberg, D-06120 Halle, Germany

**Keywords:** Amitriptyline, Histamine, Inotropy, Chronotropy, Transgenic mice, Human atrium, H_2_-histamine receptor, Phospholamban phosphorylation

## Abstract

We have previously shown that histamine (2-(1*H*-imidazol-4-yl)ethanamine) exerted concentration-dependent positive inotropic effects (PIE) or positive chronotropic effects (PCE) on isolated left and right atria, respectively, of transgenic (H_2_R-TG) mice that overexpress the human H_2_ histamine receptor (H_2_R) in the heart; however, the effects were not seen in their wild-type (WT) littermates. Amitriptyline, which is still a highly prescribed antidepressant drug, was reported to act as antagonist on H_2_Rs. Here, we wanted to determine whether the histamine effects in H_2_R-TG were antagonized by amitriptyline. Contractile studies were performed on isolated left and right atrial preparations, isolated perfused hearts from H_2_R-TG and WT mice and human atrial preparations. Amitriptyline shifted the concentration-dependent PIE of histamine (1 nM–10 μM) to higher concentrations (rightward shift) in left atrial preparations from H_2_R-TG. Similarly, in isolated perfused hearts from H_2_R-TG and WT mice, histamine increased the contractile parameters and the phosphorylation state of phospholamban (PLB) at serine 16 in the H_2_R-TG mice, but not in the WT mice. However, the increases in contractility and PLB phosphorylation were attenuated by the addition of amitriptyline in perfused hearts from H_2_R-TG. In isolated electrically stimulated human atria, the PIE of histamine that was applied in increasing concentrations from 1 nM to 10 μM was reduced by 10-μM amitriptyline. In summary, we present functional evidence that amitriptyline also acts as an antagonist of contractility at H_2_Rs in H_2_R-TG mouse hearts and in the human heart which might in part explain the side effects of amitriptyline.

**Supplementary Information:**

The online version contains supplementary material available at 10.1007/s00210-021-02065-7.

## Introduction

Histamine is synthesized by cells, such as mast cells, in many organs of the mammalian body from histidine; histamine can also be ingested with food and is transported, in part, by thrombocytes via the coronary circulation to the heart (Jutel et al. 2009). Histamine can also be synthesized in the heart (Gergs et al. 2016; Grobe et al. 2016). Histamine has positive inotropic (PIE) and chronotropic effects (PCE), which were initially described in rabbits (Dale and Laidlaw 1910). These effects can be attributed to the stimulation of cardiac histamine receptors. Currently, the effects of histamine are thought to be mediated by four receptors: H_1_ receptor, H_2_ receptor, H_3_ receptor, and H_4_ receptor (Jutel et al. 2009). There are regional differences in the actions of histamine or in the utilization of histamine receptors in the mammalian heart. For example, H_1_ receptors mediate the PIE of histamine in rabbits probably because they activate phospholipase C (Hattori et al. 1988, 1990, 1991). In the left atrium of guinea pigs, the H_1_ receptor mediates the PIE of histamine, while the PCE in the guinea pig right atrium is mediated by the H_2_R; in the guinea pig ventricle, the PIE of histamine is mediated by the H_2_R (Zavecz and Levi 1978). H_1_ and H_2_ receptors have been detected in both human atrium and human ventricle using radioligand binding (Baumann et al. 1982, 1983, 1984), antibodies, and mRNA expression studies (Matsuda et al. 2004).

**Fig. 1:**
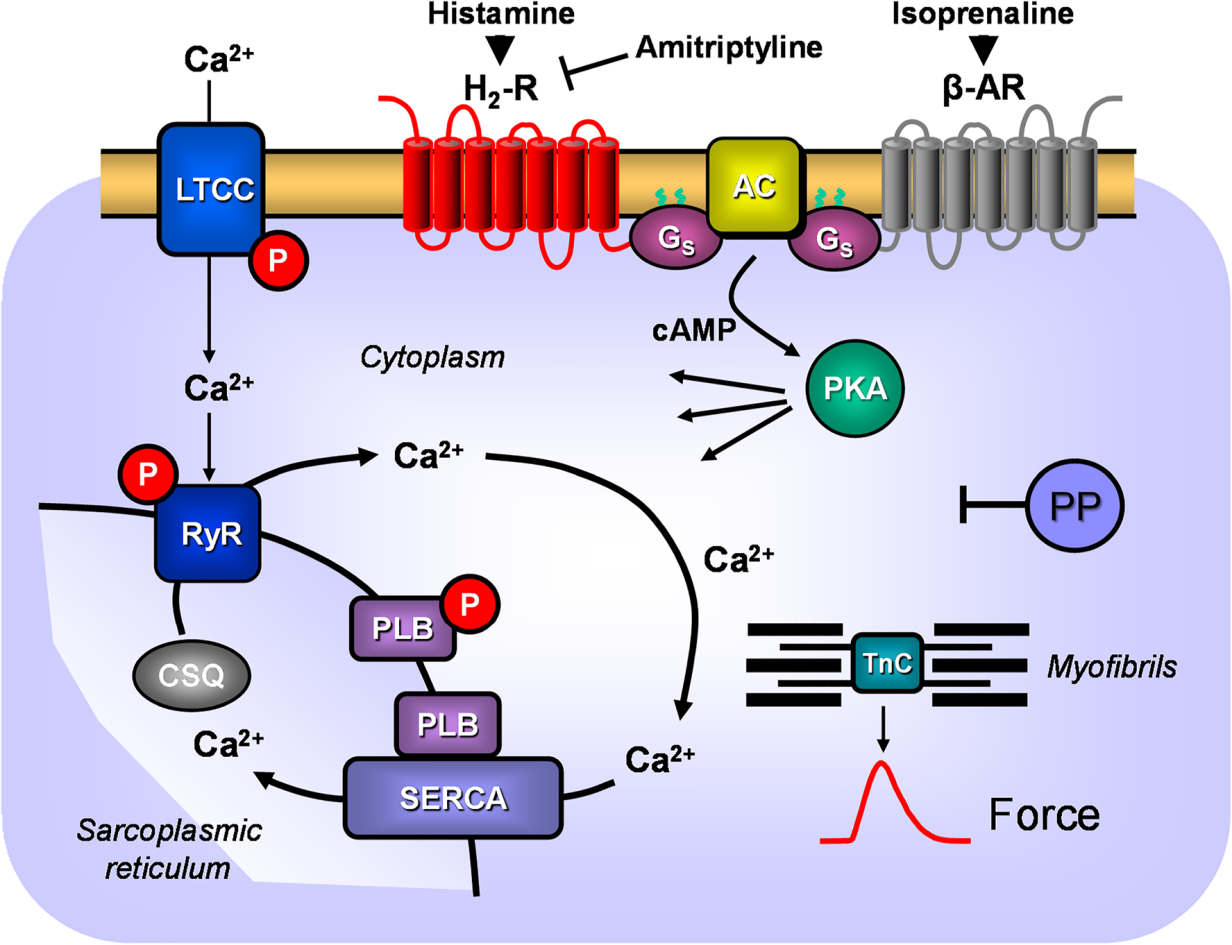
Scheme of a cardiomyocyte: histamine can bind to the H_2_ histamine receptor in H_2_R-TG and human atrium; subsequently, the activity of adenylyl cyclase (AC) is augmented in the sarcolemma via stimulatory G-proteins (G_s_); thereafter cAMP increases, and this activates the cAMP-dependent protein kinase (PKA). PKA increases cardiac force generation and relaxation by increasing the phosphorylation state (P) of the L-type Ca^2+^ channel (LTCC), phospholamban (PLB), and other regulatory proteins. Ca^2+^ initiates release of Ca^2+^ from the sarcoplasmic reticulum where it is usually bound to calsequestrin (CSQ) via ryanodine receptors (RYR) into the cytosol, where Ca^2+^ activates myofibrils and leads to increased inotropy. In the diastole, Ca^2+^ is taken up into the sarcoplasmic reticulum via a sarcoplasmic reticulum Ca^2+^ ATPase (SERCA), whose activity is higher when the phosphorylation state of PLB is elevated by PKA. The phosphorylation of proteins is reduced by protein phosphatases (PP). The H_2_R can be antagonized by amitriptyline; thus, PLB phosphorylation is not increased, force is not augmented, and relaxation is not hastened. Isoprenaline can stimulate likewise the β-adrenoceptor, which can also be antagonized by amitriptyline

Cardiac H_2_Rs have been shown to mediate the PIE of exogenously applied histamine in isolated human cardiac preparations (Genovese et al. 1988; Levi et al. 1981; Sanders et al. 1996; Zerkowski et al. 1993). The PIE in the human heart was accompanied by and, thus, may have been mediated by an increase in 3’,5’-cyclic adenosine monophosphate (cAMP) content in human right atrial preparations (Sanders et al. 1996) and the opening of L-type calcium channels (Eckel et al. 1982, Fig. 1). Hence, the mode of action of the H_2_Rs in the human heart mimics the β-adrenoceptor system in the heart (Fig. 1). Stimulation of H_2_Rs generates cAMP in the heart, which leads to phospholamban (PLB) phosphorylation (H_2_R-TG, Gergs et al. 2019b).

Interestingly, some psychiatric drugs can act as antagonists on H_2_Rs, which has been shown using radioactive labelled ligands acting on H_2_Rs expressed in insect cells by a baculovirus system (Appl et al. 2012), human brain slices (Traiffort et al. 1992) and guinea pig hippocampus and cortex homogenates (Green and Maayani 1977). One study found that the most potent H_2_R antagonist (of psychiatric drugs studied) was amitriptyline with a p*K*i of 7.18 (Appl et al. 2012). Because amitriptyline is such a potent H_2_R antagonist, it was chosen for the present study. The therapeutic plasma concentration of amitriptyline has been reported to be 255–637 nM (Baumann et al. 2004). Hence, it was reasonable to assess whether the in vitro antagonism of human H_2_Rs by amitriptyline affected cardiac contractility.

Amitriptyline belongs to the class of tricyclic antidepressants (TCAs) and is commonly used as an antidepressant drug or for treatment of neuropathic pain and prevention of migraine. Although the antidepressant effect of amitriptyline is not completely understood, it is known to block the neuronal serotonin transporter and, in part, the neuronal noradrenaline transporter. Studies have shown that, in addition to the H_2_R, amitriptyline blocks many G protein-coupled receptors, including the muscarinic receptors, α-adrenoceptors, β-adrenoceptors, and H_1_ receptors, as well as the sodium and potassium ion channels (Appl et al. 2012; Bylund and Snyder 1976; Owens et al. 1997; Pancrazio et al. 1998; Punke and Friederich 2007; Sánchez and Hyttel 1999; Stanton et al. 1993). Amitriptyline is sometimes used as a sedative, but it is also increasing the action of analgesic drugs in patients with diabetic neuropathy (Punke and Friederich 2007). Regrettably, high doses of amitriptyline are used in suicide attempts (Henry 1997) and toxic plasma concentrations > 4 μM have been reported (Preskorn and Fast 1993). The cardiovascular side effects associated with amitriptyline include orthostatic hypotension, atrioventricular conduction delays, tachycardia, syncope, lengthening of the QT interval, and subsequent cardiac arrhythmias (Teschemacher et al. 1999). Overdoses of amitriptyline have also been reported to induce a Brugada-type ST elevation (Bolognesi et al. 1997; Brahmi et al. 2007). Further examples of cardiovascular side effects after amitriptyline overdosing are summarized in Table 1.

**Table 1:** Examples of acute amitriptyline poisoning in pediatric and adult patients

Amitriptyline dosage (range and/or mean ± SD)	Amitriptyline plasma concentrations (range and/or mean ± SD and/or mean)	Main clinical symptoms (with focus on cardiovascular effects)	References
Pediatric patients			
2–97.5; 13.6 ± 17.7 mg/kg	-	Tachycardia, hypotension	Caksen et al. 2006
2.3–27; 9.4 ± 5.8 mg/kg	-	Tachycardia, hypotension, QTc prolongation	Olgun et al. 2009
0.9–41 mg/kg	0.13–16.15 μg/ml (0.5–58 μM)	Fatal intoxication, Tachycardia, arrhythmia, QTc prolongation	Paksu et al. 2014
Adult patients			
100–1000 mg	-	Tachycardia, arrhythmia, QTc prolongation	Paksu et al. 2014
3750 mg	2.39 μg/ml (8.6 μM)	Fatal intoxication, Tachycardia, hypotension, QTc prolongation, QRS widening	Bolognesi et al. 1997 (case report)
-	180–1560; 430 ± 74 ng/ml (1.5 ± 0.3 μM)	Tachycardia, hypotension, QTc prolongation, QRS widening	Langou et al. 1980
25–225; 122.4 ± 48.2 mg	32–631; 249.7 ± 149.1 ng/ml (0.9 ± 0.5 μM)	QTc prolongation	Scherf-Clavel et al. 2020
-	0.4 μg/ml (1.4 μM)	Fatal intoxication	Sunshine and Baeumler 1963
-	3.5 μg/ml (12.6 μM)	Fatal intoxications	Koski et al. 2005
-	4.3 μg/ml (16 μM)	Fatal intoxications	King 1982
-	3.3 μg/ml (12 μM)	Fatal intoxications	Stead and Moffat 1983
-	3.2 μg/ml (12 μM)	Fatal intoxications	Druid and Holmgren 1997
-	2–20 μg/ml (7.2–72 μM)	Fatal intoxications	Winek et al. 2001
-	60 μg/ml (209 μM)	Fatal, suicide or cardiac disease, defective metabolism	Koski et al. 2006
-	29 ng/ml (0.19 μM)	Coma: mixed intoxication with dextromethorphan, survived	Forget et al. 2008 (case report: poor CYP2D6 metabolizer)

Green and Maayani (1977) found that amitriptyline inhibited histamine-stimulated adenylyl cyclase activity in the brain membranes of guinea pigs in a concentration-dependent manner and had in this regard a pA2 value of 7.23. Another study of an isolated spontaneously beating guinea pig right atrium found that amitriptyline failed to reduce the histamine-stimulated beating rate, although it antagonized the PIE of histamine in the papillary muscles and had a pKa value of 6.01 (Angus and Black 1980). These results suggested that amitriptyline had a region-specific effect on H_2_Rs.

The aim of the present study was to determine whether the inotropic and chronotropic effects of histamine in atrial preparations are sensitive to amitriptyline. The atrium from transgenic (H_2_R-TG) mice that were engineered to express a functional H_2_R on cardiomyocytes (Gergs et al. 2019a) and isolated electrically driven atrial strips from the human heart were used in this study. Preliminary reports of this project have been previously published in the form of abstracts (Binter et al. 2020; Neumann et al. 2019).

## Materials and methods

### Transgenic mice

H_2_R-TG mice with cardiac myocyte-specific overexpression of the human H_2_R were generated as described by Gergs et al. (2019a) and compared with their wild-type (WT) littermates as controls. The animals were handled and maintained according to the approved protocols (I8M9) of the Animal Welfare Committee of the University of Halle-Wittenberg, Germany.

### Contractile studies in mice and human atrial preparations

In brief, the right or left atrial preparations were isolated from H_2_R-TG and WT mice and mounted in organ baths, as described by Gergs et al. (2013, 2017, 2019a) and Neumann et al. (2003). The contractile studies on the human atrium were performed as previously reported (Boknik et al. 2019). The human studies complied with the Declaration of Helsinki and followed the rules of the Ethics Committee of the University of Halle-Wittenberg (hm-bü 04.08.2005) and patients gave informed consent.

### Western blotting

Western blotting, which involved homogenization, protein content measurements, electrophoresis and protein transfer, antibody incubations, and quantification, was performed following our established protocols (Boknik et al. 2018; Gergs et al. 2009, 2019a, b). For the electrophoresis, Novex™ 4–12% Tris-Glycine Plus Midi Protein gels (Invitrogen, Thermo Fisher Scientific, USA) were run for approximately 70 min at 120 V in the NuPAGE MES SDS Running Buffer (Life Technologies, USA) using the Bio-Rad system (Bio-Rad Laboratories, USA). The proteins were then transferred to a nitrocellulose blotting membrane (Amersham™ Protran, GE Healthcare, USA) at 2 A for 2 hours and at 4 °C. The primary antibodies for PLB (Ser16, Badrilla, UK) were incubated at 4 °C overnight. To visualize the phosphorylation of the analyzed proteins, ECF staining (ECF Substrate for Western Blotting, Amersham, GE Healthcare, USA) and a Typhoon 9410 Scanner (GE Healthcare, USA) were used. Quantification was performed using ImageQuant TL image analysis software (GE Healthcare, USA), as described by Boknik et al. (2018). It is typical for phospholamban that if the homogenate of the heart is kept at room temperature, it runs as a pentameric holoprotein of about 27 kDa. In contrast, upon brief elevation in the temperature (“boiling”), it runs as a monomer at less than 10 kDa (Wegener and Jones 1984). This peculiar physicochemical property can be used to identify phospholamban by Western blotting. In other words, the bands which we depicted here as phospholamban are converted to a smaller molecular weight band upon boiling and are still recognized by the phospholamban antibody. This is depicted in a control experiment in the supplementary Fig. 1 (compare also, e.g., Fig. 1, in Neumann et al. 1994).

### Data analysis

The data were evaluated using the method previously reported by our group (Gergs et al. 2013, 2017, 2019b).

### Drugs and materials

Amitriptyline was purchased from Sigma-Aldrich (Deisenhofen, Germany). The source of the other drugs was previously reported (Gergs et al. 2013, 2017, 2019b).

## Results

**Fig. 2:**
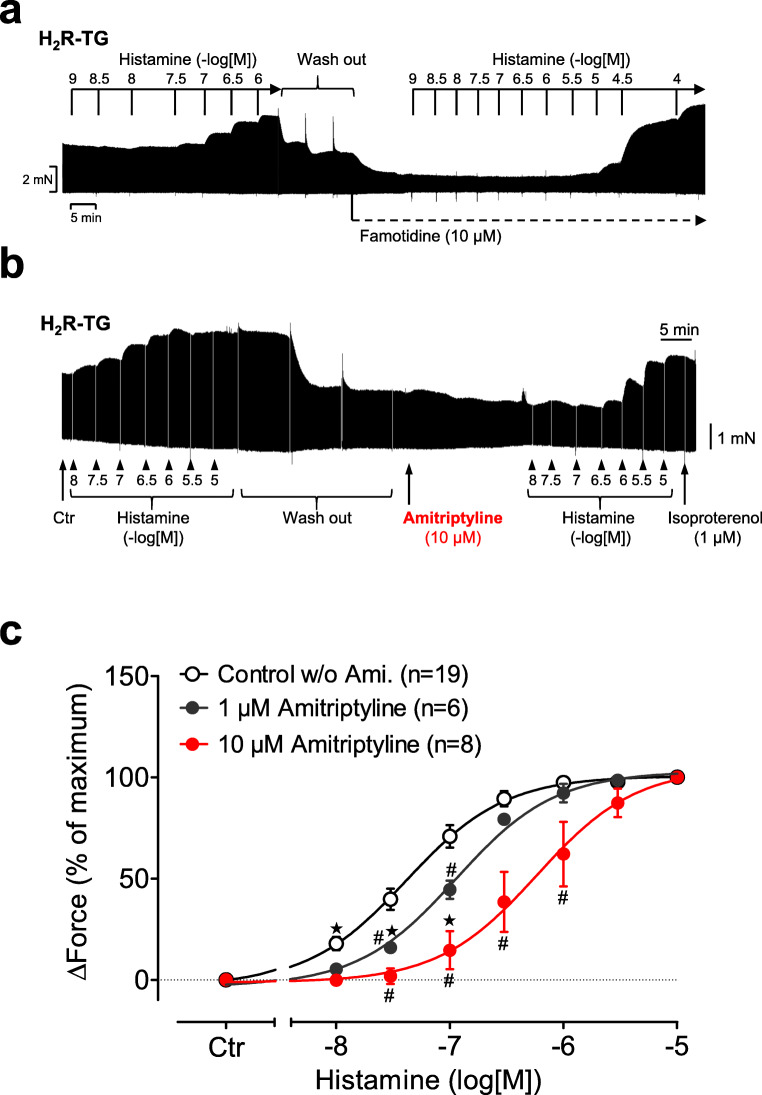
a Original recording of the force of contraction (FOC) in left atrium from transgenic mice that overexpress the H_2_ receptor (H_2_R-TG). First, a concentration response curve for histamine is shown; thereafter histamine was washed out, 10-μM amitriptyline was added, and a second concentration response curve for histamine was constructed. Finally, the β-adrenoceptor agonist isoprenaline was added. **b** Effect of histamine alone (open circles) or in the additional presence of 1-μM amitriptyline (closed circles) or 10-μM amitriptyline (red circles) on the FOC in isolated electrically driven (1 Hz) left atrium of H_2_R-TG. Ordinate: increase in force of contraction in relations to the maximum effect of histamine (=100%). Abscissa: logarithm of histamine concentration. ^★^indicates first significant difference (*P* < 0.05) vs. Ctr (= pre-drug value); ^#^*p* < 0.05 versus control w/o amitriptyline

Left and right atria of mice were prepared and after equilibration the following experimental procedure was conducted: First, a concentration response curve for histamine (1 nM–10 μM) was performed; thereafter, histamine was washed out, amitriptyline (1, 3 or 10 μM) was added, and a second concentration response curve for histamine was constructed. Finally, the β-adrenoceptor agonist isoprenaline (1 μM) was added to test the viability of the preparations (Fig. 2). The last step was necessary especially for WT preparations because histamine did not exert a PIE or PCE in the WT mice (Gergs et al. 2019b). Even though WT controls were performed, data for WT are not shown here because of the above mentioned lack of effects. A typical original recording for H_2_R-TG is shown in Fig. 2b and summarized in Fig. 2c. Our results showed that histamine exerted a PIE in isolated electrically stimulated (1 Hz) left atrial preparations from H_2_R-TG mice; the PIE was concentration and time dependent (–log EC_50_ = 7.4). To demonstrate that the histamine effects are mediated via the H_2_R in H_2_R-TG, an original recording of a concentration response curve shift for histamine by the H_2_R antagonist famotidine is presented (Fig. 2a). Amitriptyline shifted the concentration response curves for histamine to a higher concentration (Fig. 2c); the relationship between the amitriptyline and the histamine was concentration dependent. In the presence of 10-μM amitriptyline, the pEC_50_ value increased to 6.2, which was significantly higher than the pEC_50_ value without amitriptyline.

**Fig. 3:**
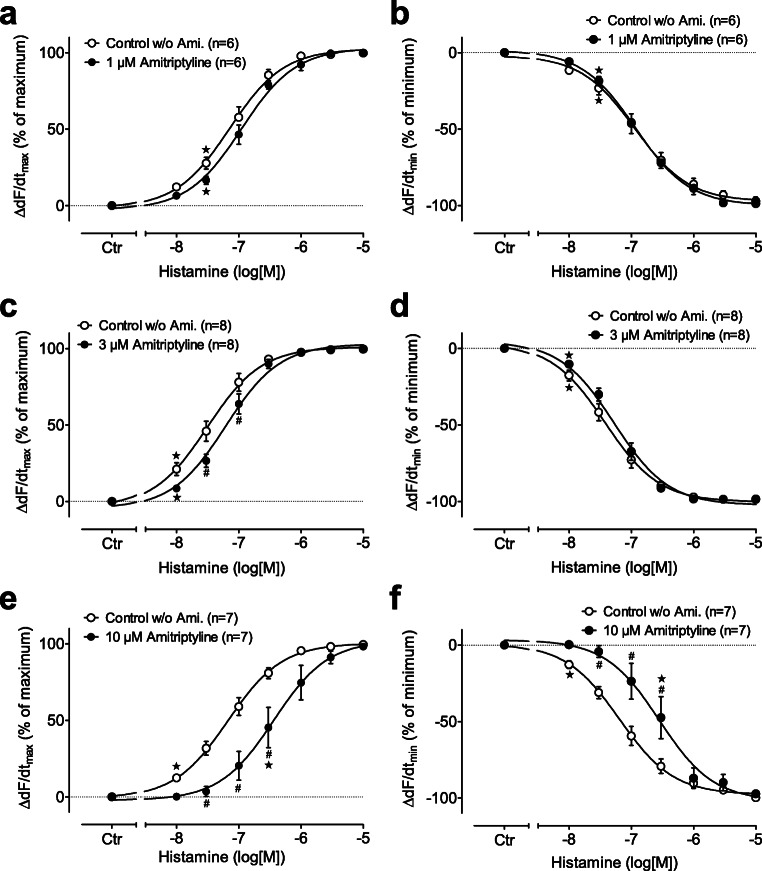
Left side (**a, c, e**): effect of histamine alone (open circles) or in the additional presence of 1-μM (**a**), 3-μM (**c**), or 10-μM (**e**) amitriptyline (closed circles) on the maximum rate of force development in isolated electrically driven (1 Hz) left atrium of H_2_ histamine receptor overexpressing mice (H_2_R-TG). Ordinate in % of maximum change of force development (ΔdF/dt_max_). Ctr = basal contraction before drug addition. Right side (**b, d, f**): effect of histamine alone (open circles) or in the additional presence of 1-μM (**b**), 3-μM (**d**), or 10-μM (**f**) amitriptyline (closed circles) on the minimum rate of force development in isolated electrically driven (1 Hz) left atrium of H_2_R-TG mice. Ordinate in % of minimum change of force development (ΔdF/dt_min_). Ctr = basal contraction before drug addition. Abscissae: logarithm of histamine concentration. ^★^indicates first significant difference (*P* < 0.05) vs. Ctr; ^#^*p* < 0.05 versus control w/o amitriptyline

Moreover, in the left atrial preparations from H_2_R-TG mice, histamine increased the maximum rate of tension development in a concentration-dependent fashion (Fig. 3). The maximum and minimum rate of tension development was not affected by 1-μM amitriptyline, but in the presence of 3-μM amitriptyline, the pEC_50_ value was reduced from the control value of 7.51 to 7.21 (*p* < 0.05) (Fig. 3). Similarly, histamine tentatively increased the minimum rate of tension development in the left atrial preparations in a concentration-dependent fashion with a pEC_50_ value of 7.42 which was reduced to 7.28 (not significant) in the presence of 3-μM amitriptyline (Fig. 3). In addition, the effect of histamine on the maximum rate of tension development amounted to a pEC_50_ value of 7.18 which was changed to 6.44 (*p* < 0.05) in the presence of 10-μM amitriptyline (Fig. 3). Similarly, histamine increased the minimum rate of tension development with a pEC_50_ value of 7.19 which was reduced to 6.55 (*p* < 0.05) in the presence of 10-μM amitriptyline (Fig. 3).

**Fig. 4:**
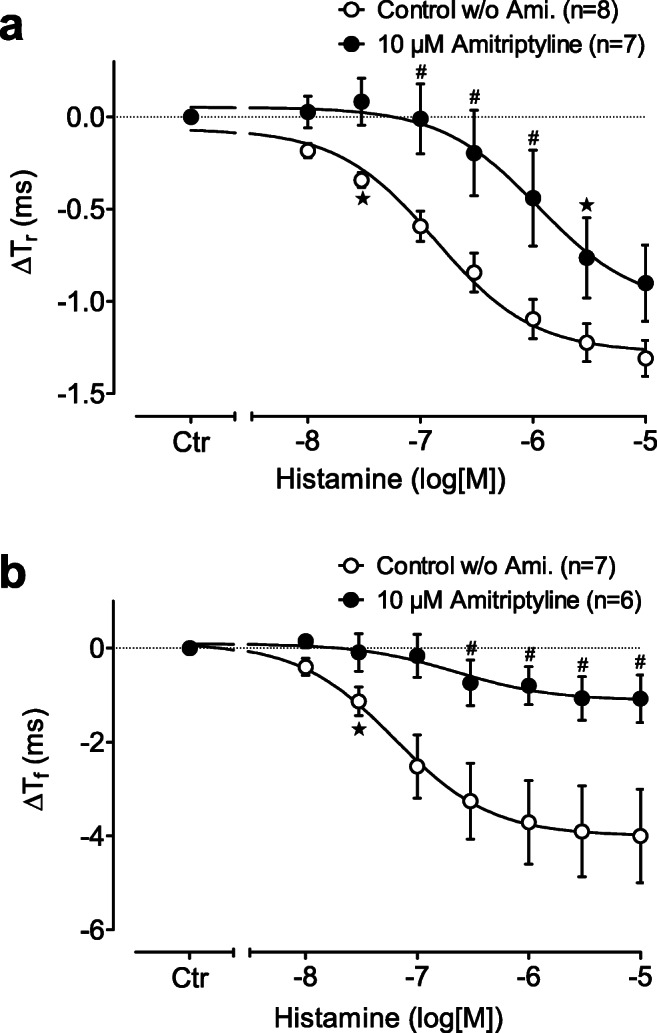
**a** Effect of histamine alone (open circles) or in the additional presence of 10-μM amitriptyline (closed circles) on the change of shortening in time to peak tension (*T*_r_) in isolated electrically driven (1 Hz) left atrium of H_2_ histamine receptor overexpressing mice (H_2_R-TG). Ordinate: change in *T*_r_ in milliseconds (ms). Ctr = basal *T*_r_ before drug addition. **b** Effect of histamine alone (open circles) or in the additional presence of 10-μM amitriptyline (closed circles) on the change of shortening in time of relaxation (*T*_f_) in isolated electrically driven (1 Hz) left atrium of H_2_R-TG mice. Ordinate: change in *T*_f_ in milliseconds (ms). Ctr = basal *T*_f_ before drug addition. Abscissae: logarithm of histamine concentration. ^★^indicates first significant difference (*P* < 0.05) vs. Ctr; ^#^*p* < 0.05 versus control w/o amitriptyline

Histamine shortened the time to peak tension (*T*_r_) and amounted a pEC_50_ value of 6.83 which was reduced to 6.02 (*p* < 0.05) in the presence of 10-μM amitriptyline (Fig. 4). In addition, histamine accelerated the time of relaxation (*T*_f_); likewise, this curve was shifted to higher concentrations of histamine in the presence of 10-μM amitriptyline (Fig. 4).

**Fig. 5:**
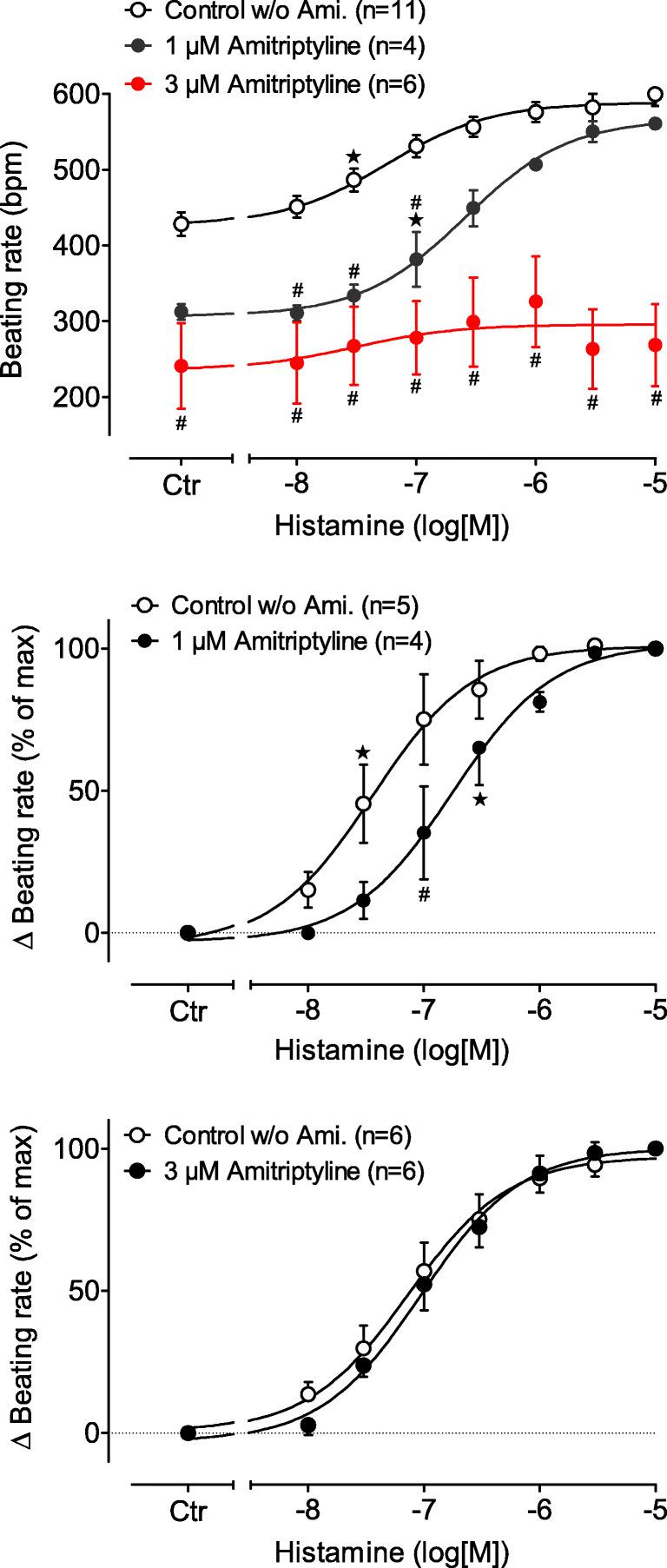
Effect of histamine alone (open circles) or in the presence of 1-μM (closed circles) or 3-μM (red circles) amitriptyline in isolated spontaneously beating right atrium of H_2_R-TG. Ordinate: beating rate in beats per minute. Abscissae: logarithm of histamine concentration. ^★^indicates first significant difference (*P* < 0.05) vs. Ctr (= pre-drug value); ^#^*p* < 0.05 versus control w/o amitriptyline

Histamine increased the beating rate in the right atrial preparations from H_2_R-TG mice (Fig. 5). The positive chronotropic effect of histamine amounted to pEC_50_ values 7.39 and shifted to 6.67 in the presence of 1-μM amitriptyline and from 7.24 to 6.36 (*p* < 0.05) with 3-μM amitriptyline (Fig. 5). We could not study the effects of 10-μM amitriptyline in the right atrial preparations because it consistently caused arrhythmias after application (data not shown).

**Fig. 6:**
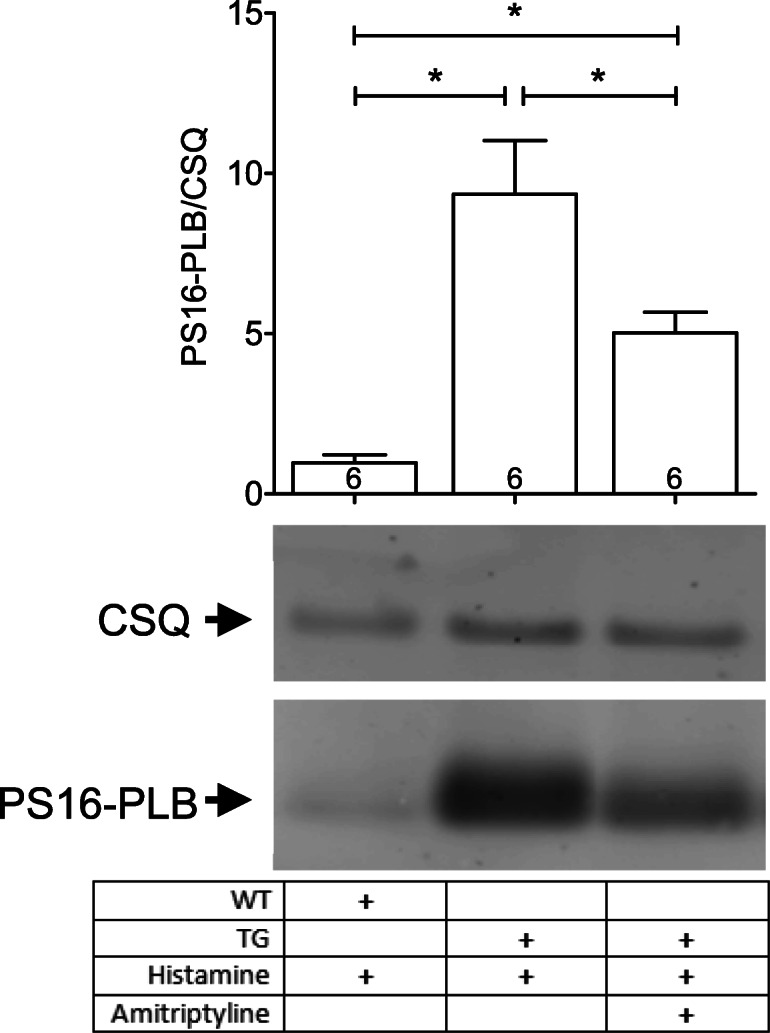
Western blot analysis of phospholamban (PLB) phosphorylation at serine 16 in Langendorff hearts from H_2_R-TG and WT mice perfused with histamine (1 μM) alone or in the combined presence with amitriptyline (10 μM). Calsequestrin (CSQ) was used as loading control. Ordinate: ratio of serine 16 phosphorylation of PLB and CSQ. *p < 0.05 vs indicated group. The numbers in the bars indicate the numbers of experiments. More details are shown in supplementary Fig. 1.

The previous data were obtained for atrial preparations from H_2_R-TG mice. For comparison, we studied the ventricular function in isolated spontaneously beating mouse hearts (Langendorff preparation). We found that 1-μM histamine exerted pronounced effects on the force of contraction in H_2_R-TG but not in WT hearts. However, this effect was nullified in the presence of 10-μM amitriptyline (data not shown). At the end of the contraction experiment, 5 min after addition of histamine, hearts were freeze clamped in liquid nitrogen and subsequently we determined whether the contractile changes in the perfused mouse hearts were accompanied by, and possibly caused by, biochemical alterations (compare Fig. 1). We noted that histamine could increase the phosphorylation state of phospholamban (PLB) at serine 16 (Fig. 6, supplementary Fig. 1). This effect was attenuated by additionally applied amitriptyline (Fig. 6, supplementary Fig. 1).

**Fig. 7:**
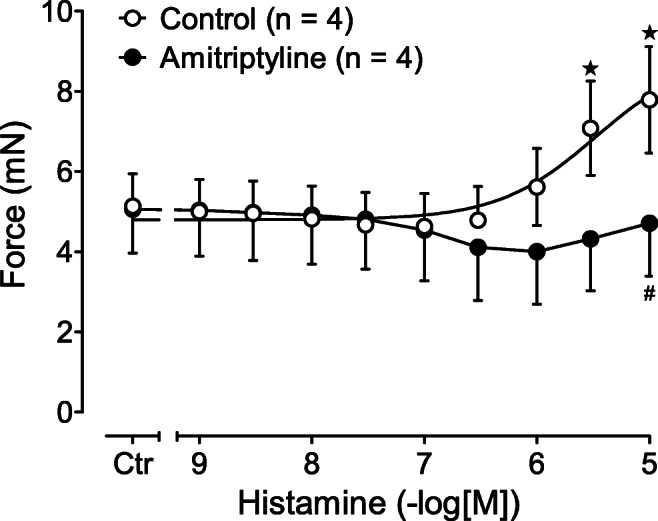
Effect of histamine alone (control, open circles) or in the additional presence of 10-μM amitriptyline (closed circles) on the force of contraction (FOC) in isolated electrically driven (1 Hz) human atrial preparations. Six preparations from four patients were used. ^★^*p* < 0.05 vs. Ctr (= pre-drug value); ^#^*p* < 0.05 versus control w/o amitriptyline

We also studied whether these contractile effects could also occur in the human heart. We found that 10-μM amitriptyline shifted the concentration response curve for the force of contraction of histamine in electrically stimulated human right atrial trabeculae carneae to higher concentrations (Fig. 7).

## Discussion

### Right atria

In previous studies, we showed that histamine will elicit a PCE in isolated spontaneously beating right atrial preparations from H_2_R-TG mice (Gergs et al. 2019b, 2020). In the present study, we noted that amitriptyline had a concentration-dependent negative chronotropic effect, which might have resulted from its known antagonism of β-adrenoceptors. However, in right atrial preparations from H_2_R-TG mice, amitriptyline was able to shift the histamine concentration response curves to the right, which is consistent with the results from an antagonism of human H_2_Rs in the sinus node of the H_2_R-TG mice. Others before us reported that amitriptyline could attenuate the PCE of histamine in right atrial preparations of guinea pig and rabbit (Hughes and Coret 1974).

We noted another result in the right atria that may merit a mention. The isolated hearts from H_2_R-TG and WT mice showed a pronounced NIE from amitriptyline and several hearts exhibited transient arrhythmias. These results agree with the known propensity of amitriptyline to cause arrhythmias. However, amitriptyline has not yet been studied in mouse hearts. One study on guinea pigs reported that 10- and 32-μM amitriptyline reduced the spontaneous beating rate of right atrial preparations, an effect that was explained by the non-H_2_R-antagonizing properties of amitriptyline; however, amitriptyline failed to shift the histamine-induced PCE in these preparations (Angus and Black 1980). In contrast, 3.6- till 10-μM amitriptyline reduced the PCE of histamine in isolated rabbit and guinea pig atrial preparations hearts and amitriptyline exerted a negative chronotropic effect given alone (Hughes and Coret 1974). We would argue that we present the first evidence that amitriptyline antagonizes human H_2_Rs in the right atrium.

### Left atria

We noticed that amitriptyline caused a small NIE in left atrial preparations from H_2_R-TG mice. This could be due to the blockade of sodium channels that has been reported for amitriptyline (Dick et al. 2007). Impairment of sodium entry would be expected to activate the sodium–calcium exchanger, which would cause sodium to be pumped into the cell in exchange for calcium. This is consistent with a NIE, which is the reason why class I antiarrhythmic agents are contraindicated in patients with systolic heart failure. A patch-clamp experiment showed that amitriptyline also blocked L-type calcium channels with an IC_50_ value of 23.2 μM, which would also in part explain the NIE of amitriptyline (Zahradník et al. 2008). It is clear that, in the left atrial preparations, histamine caused the contractile parameters, including force, the first derivative of force, time to peak tension, and time of relaxation, to shorten. This is consistent with our previously published work (Gergs et al. 2019b, 2020). Moreover, in our previous studies, we demonstrated that the effects of histamine we detect in H_2_R-TG are really due to H_2_R occupation. There, we could show that the positive inotropic effects of histamine in atrial preparations from H_2_R-TG are antagonized by the H_2_R antagonist cimetidine (1, 3, 10 μM: Gergs et al. 2019b). These data are in line with the famotidine effect presented here. The novel finding was that amitriptyline attenuated the contractile effects in a concentration-dependent way.

Another unexpected finding was that the PIE of isoprenaline, a β-adrenoceptor agonist, was also attenuated by amitriptyline. At the end of the experiment, we stimulated the samples from the WT and H_2_R-TG mice with isoprenaline to ascertain that the samples from the WT mice were responsive to β-adrenergic stimulation and, thus, were a valid control. In this experiment, we needed at least 100-μM isoprenaline to detect a PIE in the atria of the WT and H_2_R-TG mice. This result was consistent with receptor binding data that showed that amitriptyline had a β-adrenoceptor-antagonizing effect in the brain (Richardson and Hertz 1983; Sánchez and Hyttel 1999). The clinical relevance of this finding is unknown and speculative but might be determined by clinical groups in the future.

### Langendorff hearts

As mentioned in the “Introduction” section, there are regional differences in the density and coupling of H_2_Rs in the heart. We have previously reported that in the H_2_R-TG mouse model, the PIE and PCE of histamine are noticeable in Langendorff hearts and are accompanied by PLB phosphorylation (Gergs et al. 2019b). Likewise, positive inotropic ventricular effects of histamine in Langendorff hearts were antagonized by cimetidine (Fig. 4d in Gergs et al. 2019b). One interpretation of this result was that it showed that histamine used the cAMP–PKA–PLB pathway in H_2_R-TG. In the present study, we found that amitriptyline not only attenuated the PIE of histamine but also the PLB phosphorylation due to histamine, which indicated that amitriptyline can also antagonize functional H_2_Rs in the mammalian ventricle. These findings confirmed and extended previous data in guinea pig ventricle (Angus and Black 1980). For example, amitriptyline was able to shift the histamine-induced PIE to the right because of its competitive antagonistic effect on the ventricular H_2_Rs in guinea pigs (Angus and Black 1980). However, other studies showed that the guinea pig papillary muscles contain H_2_ receptors and H_1_ receptors that can elicit an NIE (Zavecz and Levi 1978). Therefore, our data are more unambiguous than data in guinea pig ventricles because there are no functional H_1_ receptors in H_2_R-TG mice (Gergs et al. 2019b) in contrast to guinea pig ventricles (Zavecz and Levi 1978).

### Human atria

We predicted that amitriptyline would also antagonize endogenous human cardiac H_2_Rs. Therefore, we performed contraction experiments on human atrial samples. Previous studies have repeatedly shown that histamine elicits a PIE in an isolated human atrium (see the “Introduction” section). In this study, we found that the PIE of histamine in electrical stimulated right atrial trabeculae carneae could be attenuated by amitriptyline. We also noted that high concentrations of isoprenaline, such as 100-μM isoprenaline, were needed to elicit a PIE in human atrial preparations when amitriptyline is present in the organ bath. This was consistent with our data on left atrial preparations of H_2_R-TG and with previous binding data (see the “Introduction” section). To the best of our knowledge, the antagonistic effect of amitriptyline in the human heart on histamine-induced effects had not been previously reported.

### Limitations of the study

We were unable to study the effects of amitriptyline in human ventricular tissue because of a lack of available samples in our institution. We await such data with interest. There is also a question about the clinical relevance of our findings. Some contractile effects were noticeable with 1-μM amitriptyline, while other effects were only significant with 10-μM amitriptyline. The highest therapeutic level of amitriptyline given to psychiatric patients was 637 nM, which is not vastly different from 1 μM. Hence, small effects of amitriptyline on H_2_Rs might be apparent in properly treated patients. In a series of fatal intoxications associated with suicidal intentions in Finland, median concentrations of 12.6-μM amitriptyline were reported (Koski et al. 2005). Therefore, we argue that our findings are of toxicological relevance. Moreover, toxic plasma levels of amitriptyline might be reached even at normal dosages. Amitriptyline is metabolized mainly in the liver by cytochromes like CYP2D6, but also by CYP1A2, CYP2C9, CYP2C19, CYP3A4, and CYP3A5 (Samer et al. 2013). In a fatal intoxication with loss of functional CYP2D6 gene, a blood concentration of 60 mg/l (216 μM) was reported (Koski et al. 2006) and drugs that impair the degradation of amitriptyline have been reported to lead to intoxication (Forget et al. 2008). The data on plasma concentrations of amitriptyline and accompanying signs of intoxication are combined in Table 1 for better reference.

In summary, for the first time, we showed that amitriptyline can antagonize the contractile effects from the stimulation of a human cardiac H_2_R. We showed these effects in a H_2_R-TG mouse model, as well as in human cardiac atrium.

## Supplementary Information

ESM 1 (PDF 273 kb)
