# Supplementary material for: Amitriptyline functionally antagonizes cardiac H2 histamine receptors in transgenic mice and human atria
Source: Naunyn Schmiedebergs Arch Pharmacol. 2021 Feb 24;394(6):1251–62. doi: 10.1007/s00210-021-02065-7 (PMC8208937; doi:10.1007/s00210-021-02065-7)
Supplement: Supplementary file 1 — (PDF 273 kb) [file 210_2021_2065_MOESM1_ESM.pdf]

Supplementary Fig. 1

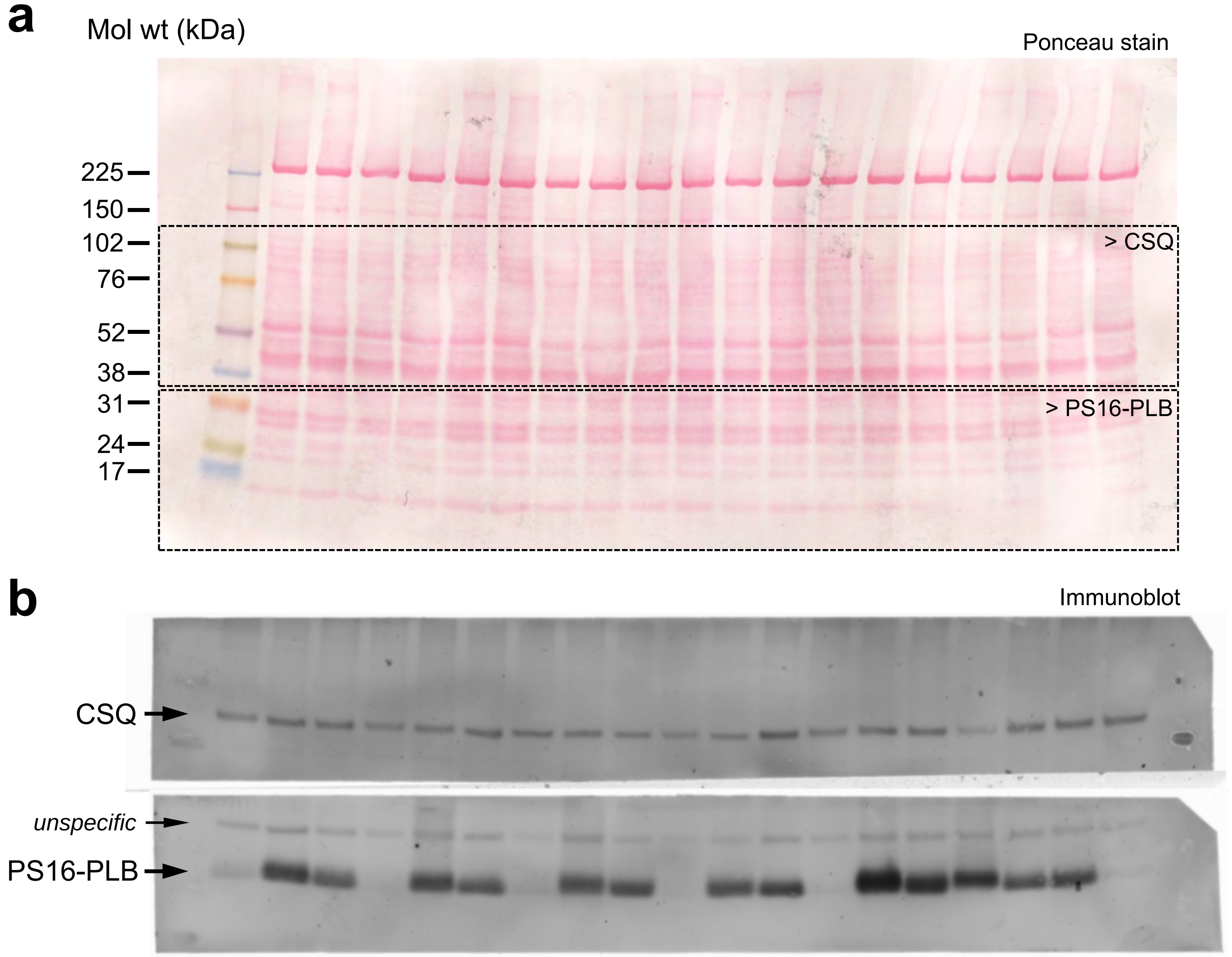

WT	+			+			+			+			+					+	+
H_2R-TG		+	+		+	+		+	+		+	+		+	+	+	+		
Histamine	+	+	+	+	+	+	+	+	+	+	+	+	+	+	+	+	+	+	+
Amitriptyline			+			+			+			+			+		+		
Isoprenaline																		+	

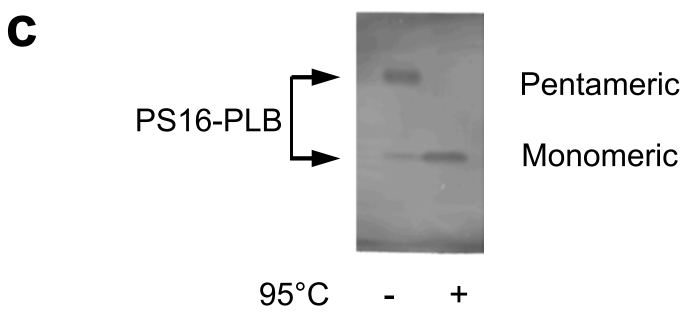

Supplementary Fig. 1
Western blot analysis of phospholamban phosphorylation at serine 16 (PS16-PLB) in Langendorff hearts from H_2R-TG and WT mice perfused with histamine (1 µM) alone or in the combined presence with amitriptyline (10 µM). Calsequestrin (CSQ) was used as loading control. Isoprenaline (1 µM) was used as positive control in WT. (**a**) The quality of the transfer was determined by Ponceau staining of the blotting membrane (Mol wt, molecular weight marker). (**b**) The membrane was cut as indicated (boxes in **a**) and the immune staining was performed. Sometimes, after longer fluorescence substrate incubation, an unspecific band appears when using the PS16-PLB antibody. (**c**) Demonstration of the PLB antibody specificity: Without heating, PLB runs as pentamer. Heating of the sample (95°C) forces PLB to the monomeric form.
